# Midwives’ Perspectives on the Adoption of a Digitalized Triage System in South African Maternity Units: Results from a Mixed-Methods Study

**DOI:** 10.3390/healthcare13091047

**Published:** 2025-05-02

**Authors:** Mxolisi W. Ngwenya, Livhuwani Muthelo, Melitah M. Rasweswe, Tebogo M. Mothiba

**Affiliations:** 1 Department of Nursing Science, University of Limpopo, Private Bag X1106, Sovenga 0727, South Africa; livhuwani.muthelo@ul.ac.za (L.M.); melitah.rasweswe@ul.ac.za (M.M.R.); 2 Faculty of Health Science, University of Limpopo, Private Bag X1106, Sovenga 0727, South Africa; tebogo.mothiba@ul.ac.za

**Keywords:** midwives, adoption, digitalized triage system, maternity units

## Abstract

**Background:** Nursing and midwifery expertise exceeds the realms of clinical and biomedical knowledge. With the healthcare system transforming towards the Fourth Industrial Revolution (4IR), midwives are expected to broaden their knowledge and skills to provide quality care through the use of digital health technologies. However, there is a paucity of studies that look at the perceptions of midwives towards these digital health technologies. **Objective:** Hence, in this case, the authors sought to investigate the perceptions of the midwives towards the adoption of digitalized triage system in their maternity units, prior to designing and implementing the digitalized system. This was undertaken to avoid imposing a huge change upon the midwives which will consequently affect the widespread implementation of the proposed system. **Methods:** A sequential exploratory research design within a pragmatic paradigm underpinned this study to gain a comprehensive understanding of the midwives’ perceptions on the adoption of a digitalized triage system in the maternity units. The qualitative phase embraced purposive sampling to select participants, and data saturation was reached at 20th midwife. Meanwhile, the quantitative phase embraced a stratified sampling technique and the sample size was 155. The mixed methodological analysis was conducted using a case-comparison analytical strategy. **Results:** The study revealed that the midwives perceived that the adoption of a digitalized triage system would improve their skills and enhance positive health outcomes for the patients. However, they were concerned with implementation challenges such as the availability of resources and network connectivity. **Conclusions:** The findings suggested that midwives in this modern era are accepting of digital health interventions as they perceive them to be useful. However, the digital health intervention was also perceived to possibly be affected by external factors such as digital illiteracy, lack of resources and internet connectivity failures. Therefore, there is a need for the development of guidelines and a conceptual framework dedicated to facilitating the widespread implementation of digital triaging in maternity units in South Africa.

## 1. Introduction

Nursing and midwifery expertise exceeds the realms of clinical and biomedical knowledge. With the healthcare system transformation towards the Fourth Industrial Revolution (4IR), midwives are expected to broaden their knowledge and skills to provide quality care through the use of digital health technologies [1,2,3]. According to Makhene, in this new era of nursing, with the challenging healthcare settings, new technology advancements and ever-growing demands of patients for appropriate care, midwives need to become critical thinkers. Technological advancements instill change in the roles and responsibilities of midwives. As critical thinkers, midwives are supposed to make effective decisions and solve complex problems to achieve the optimum health of their patients [4]. One of the roles involves the use of digital health innovations.

Digital health innovations have now become an area of interest in the healthcare industry to bridge the gap from suboptimal to optimal quality care. The use of digital health innovations in maternal health has been associated with positive outcomes. Several authors have alluded that the use of technological innovations has significantly improved maternal outcomes and patients’ satisfaction of healthcare services delivery [2,5,6,7]. To achieve this effect, some of states have proceeded with a transition from traditional models of care to the use of digital health innovations [6]. The existing literature highlights that the transition to digital health innovations in the African context seems to be sluggish. However, it is also suggested by existing evidence that digital health technology is a platform that improves the quality of care and timely provision of optimal care, particularly in maternity care. The use of digital health technology engages women in their care and supports women and their families. Furthermore, it promotes good maternal health practices and facilitates quality maternal care [2,5,8].

This current study looks at the digitalization of a triage system for midwives. It is supported by evidence that approaches used for the triaging of women in maternity units has evolved, involving the use of digital technological triage models evolving from the traditional healthcare triage system to a digital format. Triaging practices have seemingly expanded as other clinical institutions rely on the use of digital platforms in triaging within their emergency and maternity units and have cut ties with the traditional healthcare systems. For example, Wunsch et al. explored the use of a Semantic-Based Model for emergency department triage and reported positive results. Nurses expressed that the mobile triage tool was effective and improved the triage process [9]. Existing evidence shows that the use of digitalized triage systems improves patients’ outcomes. This is achieved through the use of advanced artificial intelligence algorithms that can predict the severity of illness and emergency. A scooping review by Ngwenya indicated that the use of digitalized triage systems has also been associated with improved confidence and competence among healthcare professionals [10,11]. Engeltjies et al. and Choosri et al. indicated that digital triage is associated with improved and accurate patient diagnosis. Moreover, the authors indicated that digital triage improves patient satisfaction with the emergency healthcare services provided [12,13].

Surprisingly, in South Africa (SA), there is limited evidence about the digitalization of triage systems in maternity units. The growing practice of using digital health innovations in maternal health in SA has been predominantly linked to health promotion and prevention digital innovations, such as mom-connect and Pregnancy Plus. There is a paucity of research that investigates the adoption of digital technologies in the management of obstetric emergencies. Therefore, this presents an opportunity to address the existing substantial gap in the SA context. It is suggested by the Technology Acceptance Model that before designing and implementing digital health innovations, the perspective of the end users about the idea should be determined because the decision to reject or accept digital innovations remains an open question. Based on the TAM, the authors thought it would be advisable to look at the perceived usefulness of the digitalized triage system before co-designing the system with the midwives [14,15]. This was similar across other theories such as TAM2 and the unified theory of adoption and use of technology. These theories suggest that the behavioral intentions and perceptions of use among users are influenced by various factors such as subjective norms, quality of output, job relevance and result demonstrability [16,17]. Thus, this paper investigates the perspectives of midwives on the adoption of a digitalized triage system in South African maternity units. This contemporary study lays the foundations for a potential co-designing of a digital triage system to improve the skills of midwives in triaging pregnant women in maternity units of South Africa. Through the digital midwife-led triage system processes, good maternal and child health and well-being (SDGs 3.1 and 3.2) could be achieved.

## 2. Methods

### 2.1. Research Design

This paper forms part of a larger Doctor of Philosophy project that investigates three varying phenomena to design and develop a digital midwife-led triage system. A sequential exploratory design was adopted within a pragmatic paradigm to comprehensively gain an understanding of the perceptions of the midwives on the adoption of a digitalized triage system. This research approach encompassed the components of both qualitative and quantitative research methods independently at a specific point and combined at another point for the purposes of breadth and depth of understanding of the topic under study [18]. The mixing of qualitative and quantitative research strengthened the study by enhancing the validity of the study and further offered the author significant opportunities to gain a deeper understanding of complex philosophies and perceptions concerning a potential digitalized triage system. The qualitative research findings at some point informed the development of the questionnaire tool for the quantitative phase [18,19]. The author has undertaken this research design approach in this study with the hope in mind that the mixed-methods approach will advance scientific understanding of the phenomenon under study and fill in the existing substantial scientific gaps.

On the other hand, the pragmatism paradigm enhanced the functionality, integrity and accuracy of the research approach. Moreover, it focuses on practical problem solving of social problems by emphasizing communication and mutual shared meaning [20,21]. In the context of the study, the pragmatism paradigm enabled the author to emphasize the shared meaning and perceptions of midwives on the adoption of a digitalized triage system in the labor wards through the complementarity of the qualitative and quantitative research results. This study is presented over 3 phases, namely, qualitative, questionnaire development and quantitative phases.

### 2.2. Study Setting and Sampling

The study was conducted in 10 district hospitals of Mpumalanga province. The district hospitals were dispersed all around the three districts of Mpumalanga province, namely, Ehlanzeni, Nkangala and Gert Sibande. The target population was an estimated 300 midwives working the maternity units of district hospitals. In the qualitative phase, purposive sampling was employed to purposively select midwives working the maternity units of the district hospitals. The sample size was directed by data saturation, which was reached with the 20th participant. However, in the quantitative phase, a stratified random sampling method was embraced to provide all the participants from the district hospitals with equal chances to participate in this study. The sample size was determined to be 171 using the Slovin formula. However, only 155 questionnaires were returned.

### 2.3. Recruitment and Data Collection

In the qualitative phase, during the recruitment process, the first author as the facilitator of the interviews introduced the conceptual meaning of the concepts like digitalization and triage. However, before that, the first author engaged the participants in an in-depth conversation to understand their understanding of what triaging is in maternity units. These findings are depicted in the article published in another journal [22]. An idea of a digitalized triage system was then proposed to the midwives and thereafter their perspectives of a digitalized triage system were investigated. The data were collected using semi-structured interviews which were recorded with audio-tapes upon the midwives’ consent. The interview process went on until data saturation was reached, with the 20th midwife. Each interview lasted for about 30–50 min. The data collection took place from May to June 2024.

Meanwhile, the quantitative phase took place after the data collection and analysis of the qualitative phase, from August to October 2024. A 13-item self-developed questionnaire was used for data collection. The items in the questionnaire were derived from the qualitative phase. Ordinal scales in the form of the Likert scale were used to measure the perceptions of the midwives’ variables. Using the Likert scale, it gave the participants a chance to settle on a choice for their degree of agreement with the provided statement. The provided Likert scale ranged from disagree to agree. Nonetheless, since the participants were already prepared and briefed on data collection during qualitative data collection, the adopted research process was explained to them. Thereafter, the first author proceeded with data collection with the assistance of three field workers (two female and one male). The author and field workers administered and supervised the full completion of the questionnaires by those participants who were willing to participate in this study.

### 2.4. Data Analysis

The qualitative data were analyzed following the steps of the thematic analysis. The authors followed the analytical process suggested by Naeem et al. [23]. The authors dived deep into the transcripts to first familiarize themselves with the raw data and read. This enabled the authors to understand the raw data to discern initial themes and important data. The authors identified recurring patterns and terms to encapsulate the midwives’ perspectives on the phenomenon under inquiry. Initial codes were then identified and selected. Thereafter, the authors proceeded to convert the raw data into meaningful insights by developing themes and conceptualizing the similar codes and categories [23]. All the authors conducted their individual analysis and reached a consensus on the themes that emerged. Any disagreements among the authors during analysis were solved through open communications and all discrepancies were discussed. Quantitative data were analyzed using a Statistical Package for Social Sciences version 29.0. Descriptive statistics were employed with the assistance of the university biostatistician. The descriptive statistics reflected the frequencies, means and percentages of the categorical variables. Lastly, the Cronbach alpha was measured to examine the internal consistency and reliability of the questionnaire. The overall Cronbach alpha of the 59-item questionnaire was 0.90. For that reason, it was concluded that the questionnaires were excellent and this confirmed the reliability, dependability and stability of the items employed in the instrument of this study, as suggested by Shrestha [24].

A cross-case comparison mixed-methods analytical strategy was adopted in this study to connect, combine, compare and merge the qualitative with the quantitative data. This mixed-methods analytical strategy allowed the highest level of extension of creating blended variables and it consolidated the data on qualitative and quantitative phases through building holistic, internal coherent profiles that were used to test or expand upon the qualitative or quantitative derived themes for comparison purposes [25,26,27]. Furthermore, a joint display was used for visual presentation to epitomize the integration of the qualitative and quantitative data for comparative and integration purposes to draw out new insights beyond the data for the qualitative and quantitative data as single research methods. Noting the nature of the mixed-methods design, building and merging integration techniques were used as the integration techniques. The building integration technique is an approach in which qualitative findings are used to build on the development of the quantitative data collection tool. Since the authors adopted an exploratory sequential research design, achieving the building technique was inevitable [27,28,29]. Separately, the authors first collected the qualitative data through interviews and analyzed the data using thematic analysis. Thereafter, the themes, subthemes, and direct quotes from the qualitative data were used to build onto the quantitative strand.

### 2.5. Methodological Rigor

The rigor of the qualitative strand was ensured by following the five general elements to ensure trustworthiness, namely credibility, transferability, confirmability, dependability and authenticity [30]. The first author spent a long period of time and engaged in depth with the participants to understand them better and gain insight into their perceptions. This enabled the authors to ensure credibility. The authors further ensured dependability and authenticity by faithfully reflecting the participants’ feelings and tones through data interpretation of the field notes. Moreover, to address fairness during authenticity, the participants were all provided with informed consent detailing the study process and purposes and were all interviewed using the same interview guide. The data were coded by all the authors to ensure confirmability [30]. In the quantitative strand, validity and reliability were ensured via various processes. This is detailed in the Development of the Questionnaire section.

## 3. Results

In this section, the qualitative findings, development of the questionnaire, quantitative findings and mixed-methods findings are presented.

### 3.1. Qualitative Phase

The qualitative phase generated two themes and eleven sub-themes. The themes were named perceived benefits and effects of digitalization of a triage system, and perceived difficulties with the use of digitalized triage system. Table 1, below, shows the themes and subthemes that emerged.

#### 3.1.1. Theme 1: Perceived Benefits and Effects of Digitalization of a Triage System

Upon the realization of the impact of digital health in some aspects of healthcare, the midwives were engaged in a conversation about their views and perceptions on introducing a digitalized triage system in the labor wards. The midwives discussed in depth their perceived benefits and effects digitalizing of the triage system. Among the perceived benefits were reduced workload, learning opportunities, improved patient care and information loss prevention.

##### Subtheme 1.1: Perceived as Easy to Use

It really is a new dawn in the maternity units of South Africa. The midwives sang out positive praises towards the digitalization of the triage system. The first impressions were that digitalization is the initiative that they have been longing for and it is definitely a beautiful initiative. With a glimpse of a smile in their face and a show of excitement and amazement with widened eyes, the participants said

“*It seems like a very interesting initiative, yeah, I’ve been longing for one… so I think digitalization will be very, very helpful with the time*”

**P11**

“*It would be much, much easier. I just wish it could be like that, because where I come from, I went there for six years. Yeah, so I’ve seen, and when I went there, when I came, when I arrived there, they were still, they had all the system, but when I left, they were now paperless. Yeah, so, yeah, I think, I think it would be, it would cause much difference*.”

**P3**

“*I think that the digitalization will make us to work easily because you will no longer refer to papers which takes more time so instead when you have your file you’ll just go to the computer and type in whatever information you want to keep record of. So, it’s going to be easy for us*.”

**P6**

##### Subtheme 1.2: Reduced Workload and Paperwork

The midwives expressed that the transition to a digitalized triage system will reduce the workload and paperwork. The main reason was that the midwives mentioned they are currently overwhelmed with paperwork. This was supported by the following assertions:

“*It would be okay because it would cut off the paperwork time because we do get tired with handwriting files. So, when we get used to it, I think it’s going to be okay*.”

**P18**

“*… and also reduce paperwork and optimize staff resources and then the digital it will provide better data management because we will be having digital system, meaning we will need less paperwork*”

**P9**

On the workload reduction, one of the midwives said

“*So, it would help us minimize our work actually. Because this side has a lot of work like for instance writing the file of a patient, even where I’m from we don’t write a file this way*.”

**P4**

##### Subtheme 1.3: Training and Professional Development Opportunities

In the realm of the perceived beauty of a digitalized triage system, the midwives further expressed that the presence of a digitalization of triage system would provide them with learning and professional development opportunities. The midwives viewed the introduction of a digitalized triage system as a learning curve and an opportunity to improve their skills and learn new things. This was corroborated by the subsequent quotes:

“*…it would help us also to develop our skill, our skills in some sort of way, yeah…*”

**P11**

“*…also, maybe it will also be a way for us to learn, because we won’t be stuck in an old system because we are lazy to learn; so, it is part of learning*”

**P19**

A probing question was posed to one of participants:

“*So, what other opportunities do you think it might come with?*”

With enthusiasm and amusement written all over their face, the participant responded

“*Yes learning, we will get to learn new things.*”

**P5**

##### Subtheme 1.4: Time Efficiency and Time Management

The midwives expressed that digitalization in the labor ward is a very interesting initiative. The midwives further expressed that use of the digitalization will definitely be time-efficient. As paperwork time will be reduced, they will have more time on their hands to interact with patients. The participants said

“*It seems like a very interesting initiative, yeah, I’ve been longing for one, because this paper thing, paper thing, yeah, it’s very, very much concerning, it’s very, it’s, it wastes a lot of time, so I think digitalization will be very, very helpful with the time*”

**P11**

“*I think that it is going to help in reducing the time that we spent on paperwork.*”

**P15**

One of the midwives with experience on a digitalized system from private industry was asked *“How was it for her?”*, and her response was

“*As a midwife, you know, it’s, I just wish it could make a lot of difference. Yeah, because while I’m busy, when I have…I put you on the machine, which does everything, and when I’m entering you on the system, I could also attend to another patient. Yeah, like it’s, it could, it’s time management*.”

**P3**

##### Subtheme 1.5: Safeguard and Prevent Information or Records Loss

The midwives identified that the crucial element in digitalizing a triage system is that it will prevent patient information loss. Their concern is that with the already existing traditional filing system, some of the patient’s clinical information gets lost and sometimes the whole clinical record is misplaced or lost, predisposing them to litigations.

“*I think it will prevent loss of records, because sometimes you found out that patient records are lost by that time you already wrote down everything in the records. But at the end of the day, you look for the file and is nowhere to be found. Now there’s a litigation but you can’t find the file anymore*”

**P2**

“*Oh, which means, I think sometimes it is going to help us not lose information. Even if a patient comes back after a year or 2 years. So, when she comes back, maybe let’s just say the patient was done a previous C/section. So, you know that if a patient is previous C/sections we categorize her as high risk. So, if ever that information is documented and well kept. The advantage would be that we’ll not lose information*”

**P12**

“*What’s beautiful about it is that information gets to be stored for a long time. Unlike papers because papers and files can be lost or stolen at any time. But in the computer, the information gets stored there for a long time.*”

**P19**

Related to the safeguarding of patients’ clinical information, the midwives expressed that with the digitalized system, the information will not be lost and it will be stored in the system. The storage of the information in the system is interrelated to the enhanced accessibility of patients’ information, particularly when they will be visiting the clinical institution in the near future. Hence, they perceived that the use of digitalized system will address their existing concern of clinical record discrepancies such as loss of files.

##### Subtheme 1.6: Improved Patient Care and Outcomes

In their views of triage system digitalization, the midwives dwelled on its significance in improving patient care. The midwives underscored that the digitalization process will definitely bring about improved health outcomes for both baby and mother. The midwives highlighted that the use of a digitalized system will improve patient safety through the identification and detection of problems early and alert them in time.

“*It will improve quality care… it will bring positive results for patients*”

**P7**

“*The introduction of digital health in maternity unit has the potential to revolutionize the industry… offering numerous expected benefits for mothers and healthcare providers as well the overall healthcare systems… So, it can also access or improve access to patients… uh especially the ones in rural areas they can access to maternity care through digital. It can also make patients to be more involved in their health. So the digital tools, can empower the women to play an active role in their health…so to the health providers it will make it easier for them to collaborate effective with other multidisciplinary team healthcare professionals… also it will reduce errors, isn’t we will be doing everything digital and it might also improve patient safety…Most importantly is will provide us with viable data to improve maternity care and outcomes, because we will be digital and it will give us information on how to improve our quality care and have better outcomes. Overall, I mean the introduction of digital in maternity units it has a potential to enhance quality care… and have accessibility and patient centeredness leading to better health outcomes, the mothers will be okay as well as their babies.…it will improve patient care and there will be timely interventions for our patients, there won’t be delayed interventions… it will lead to better outcomes for the babies and mothers, also it assist provide personalized care…as well it will improve patient safety because the digital tool can identify health risk in our patients early and then alert us so that we can also act accordingly and provide patient care prevent complications and harm to our patient, because it would have already identified the potential problem or risk in time and we can manage early… also it will guide us to make improvement plans, let’s say if there’s somewhere we are lacking, so it alert us early, so we can come up will improvement plan*”

**P9**

“*It will very good, patient care would be much improved because we can now identify the problems early and forward them to the other system very quickly than relying on the papers and stuff*”

**P8**

#### 3.1.2. Theme 2: Perceived Difficulties with the Use of a Digitalized Triage System

Despite the feeling of excitement displayed by the perceived benefits of digitalized triage, the participants’ feelings were conflicted as they raised concerns and perceived difficulties that would be associated with the implementation of the digitalized triage system. Among those difficulties were digital illiteracy, resistance to change and finances to sustain the digitalized triage system.

##### Subtheme 2.1: Concerns About Network Connectivity and Technical Failures Related to Power Outages

In spite of the perceived benefits and effects of a digitalized triage system, the midwives raised a concern of network connectivity related to loadshedding and the affordability of data. They felt that although a digitalized triage system would definitely be a good initiative, they could not disregard the current existing problems that might impact its effective implementation in the labor ward. The worries about network connectivity were supported by the following assertions:

“*…another challenges the digital will bother us when it comes to loadshedding, our electricity it on and off every now and then…Also the backup in our institution is malfunctioning and it delays sometimes, so that it will affect the provision of care particularly when there’s loadshedding and network connectivity issues…*”

**P9**

“*…I think there’s loadshedding as well; there are less disadvantages than the advantages.*”

**P11**

To further explain their concerns regarding network connectivity, midwives related a lack of data among midwives to contribute to the network connectivity issues. They opined that the digitalization system will definitely need data to function. This was supported by the following narratives:

“*I think challenges would be maybe if there’s no network, or we don’t have data, yeah, we don’t have data or network to access the system*”

**P11**

“*I think the challenges would be that someone else will tell you that I didn’t use the app because I didn’t have data*”

**P1**

In addition, one of the midwives raised an essential concern of technical failures associated with the use of digital systems. The midwife expressed a concern involving running out of space in the system and the possibility of it being hacked by viruses.

“*…maybe if it runs out of space, or there’s any viruses that might hack into the system, or then you end up losing the information of the patient, but other than that…*”

**P11**

##### Subtheme 2.2: Resilience and Resistance to Change and Digital Illiteracy Among Veteran Midwives

In the realm of digitalizing a triage system, the midwives anticipated that with a digitalized system, resistance and resilience among the midwives might be a huge issue. The midwives feared that the resistance and resilience to digital triage use may mostly happen among older midwives, as they might not be as digitally orientated and digital literate as the younger generations.

“*…especially older people, they are resistant to change. You know, yeah, I think the challenge will be that. Yeah, because I’ve seen it, that there are cases where things must be done digitally. They’ll say, no, we still write. You know, even now they’ve introduced this online, like a, what do you call it? The e-filing. No, not e-filing thing, but e-salad or what, what do you call it? The e-leave thing. Yeah, e-leave thing. Still, they want us to fill out, they fill the form, so what’s the use of introducing that e-leave? Yeah, so I think the challenge will be the older generation. Yeah, I think that will only be the challenge.”*

**P3**

“*Then the old nurses that will resist to change and now we have to go digital… some don’t have smart phones, although most of us have them… you find that even that she has, she doesn’t really know how to use it. So, to open the digital system might be a problem to her. So now we won’t know if you going to have a lot of old nurses in one unit what is going to happen*”

**P10**

On digital illiteracy, another midwife said

“*Even though it going to be challenge for people who cannot use technology, but it’s going to at least go faster with regard to the results, it will go faster because even patients are going to receive the results faster than using papers.*”

**P8**

On the topic of digital illiteracy, the veteran midwives expressed their concern of using digitalized triage system. With a serious face, the midwives said that they could not do it. The midwives related their inability to use technology to their age and experience of life before technologically advanced times. The use of a digitalized system would prove to be difficult for them.

“*Myself because I’m old so I can’t. Myself with a phone or computer are not friends, am not good with those digital things. So, I wouldn’t agree…*”

**P1**

“*Remember us we were born before technology so we are not familiar with these digital things. So, I don’t know…*”

**P2**

##### Subtheme 2.3: Concerns About the Finances and Cost to Sustain the Digitalized Triage System

The midwives reported that although a digitalized triage system is a good thing, they could not help but worry about the costs and finances to kickstart it and keep it running. Among the concerns about funds and costs were training costs for the new innovative development and sustainability and maintenance of the existing system. These were supported by the following connotations:

“*…now the patient will be managed on time. So, even if I think it’s expensive. It’s expensive, but it’s good…*”

**P8**

With worry in their voices, other midwives said

“*But isn’t go cost us a lot of money to train the midwives and so everybody can use that system*”

**P2**

“*…to create such an app is very expensive. Okay. Yes, to create an app is very expensive and it needs maintenance because you need to upgrade your app as well. Remember, the versions must be upgraded. They will say this is an old version because now there’s something that is added into the app, you must upgrade your app. I don’t know how to explain it, but it will make much difference.*”

**P3**

##### Subtheme 2.4: Concerns of Training Midwives About Reformed Digitalized Triage

In addition to worries about finances, the midwives went on to relate their concerns about the need for training, as a digitalized system is a relatively new innovative within their clinical facilities. The midwives’ concerns were related to number of factors, among which was digital literacy.

“*…so now the challenge will be that a training for midwives and staff already working in maternity before we digitalize… because you cannot say now everything on paper should be paperless, they need training. They don’t have knowledge and are they computer literate, we are not… so we are not ready unless we undertake training.*”

**P2**

“*Before we get to that, I think we’ll have to attend training. We need to acquire knowledge for using such triaging system because not everyone is computer wise.*”

**P4**

“*Some other nurses like the old ones are used to writing down in papers so they might not be able to understand this digitalization so they’ll need sort of like training on how it works*”

**P17**

“*As long as there will be a training on how to use it, everything will be fine*.”

**P15**

“*So, I think in our institution before we can start will the digitalization, we need to in-service our healthcare providers, we need to do training…there are some nurses who can’t use computers, so yeah we need training so that everyone could be familiar with this digital thing…*”

**P9**

##### Subtheme 2.5: Availability of Resources

Availability of resources was also described and discussed as a potential difficulty in the use of a digitalized triage system. A digital triage system cannot be implemented without adequate resources. The midwives underscored that the availability of resources for digitalized triage systems is a major concern. The shortage of resources was a prevalent theme in this study. The midwives related their perceptions of the introduction of a digitalized triage system to their current struggles with resources. As they strongly iterated, it might not work because even now, they lack equipment and when the implementation of a digitalized system comes, they will be told about budget as they normally are. This was supported by the following subsequent assertions:

“*Challenges will that won’t have laptops, and tablet. They will tell us about the budget. As always*.”

**P8**

“*Eish… I think in some other hospitals is going to be good, but as for this one of ours I doubt because for instance even now we’re failing to have enough equipment we only have three machines so I think even if we are to implement the digital triaging system probably, we’re going to have a shortage of the necessary computers to use for triaging*.”

**P4**

### 3.2. Development of the Questionnaire

The items in the questionnaire were developed based on the themes that emerged and the direct quotes of the participants. Table 2 displays a joint display delineating qualitative themes and subthemes in the developed questionnaire. The questionnaire underwent various processes to ensure its validity and reliability. During the item generation phase, as previously noted, the author relied on qualitative data and engaged in several iterative rounds of reviewing the qualitative findings and relevant literature. Additionally, the first author held ongoing meetings with the university biostatistician and regularly consulted with the co-authors (L.M, M.M.R and T.M.M) throughout this process. Through this process, the university biostatistician provided his insights and expert opinions. The process of building the items from the qualitative phase is depicted in Table 2.

On the same wavelength, the authors achieved content validity by reviewing the relevant literature extensively to identify the critical components for the questionnaires; however, the majority of the questionnaire items were mainly based on qualitative results. Furthermore, a group of field experts were invited and consulted via email to assess and analyze the questionnaire to determine whether the content covered was useful or of no important or relevance to the phenomenon under inquiry. A Google survey was sent to a total of eight experts; however, only three responded. The experts ranged from researchers to experienced midwives and midwifery specialists. The questionnaire was given to the experts, with a concise scale range of “Not relevant” to “Relevant”. The results from the experts were then captured and analyzed with Microsoft Excel^TM^. The analysis of the Item Content Validity (I-CVI) was calculated by adding somewhat-relevant and relevant responses from the experts, and this led to the calculation of the Scale Content Validity (S-CVI). A 59-item questionnaire was then developed. However, only 13 items were representative of this study objective, out of the 59 items. The overall calculated Scale-Content Validity Index (S-CVI) and Universal Agreement (UA) of the complete questionnaire were 0.95 and 0.85, respectively, indicating excellent content validity, as suggested by other scholars [19,31,32]. Any CVI less than 0.78 as indicated by several authors was revised and modified with the assistance of the co-authors. Overall, the item analysis revealed that all the items in the questionnaires were valid and relevant as rated by the selected experts. In addition to the expert validation, the questionnaire was sent out to at least 30 participants in diverse hospitals to check for any errors in the questionnaires and that the items in the questionnaires were relevant to their field of practice, as well as to check the reliability of the questionnaires. Only 25 participants responded. The filling out of those questionnaires by the participants was not performed under duress; however, the author provided the contact details for the participants during the filling of the questionnaire to address any queries and give clarity when needed. Thereafter, the questionnaires were reviewed and rectified based on the pilot study outcomes. For instance, the author corrected spelling errors noted on the survey items and removed the abbreviations from questionnaires, as the researcher noted they caused confusion among the participants.

### 3.3. Quantitative Phase

The findings in Table 3 show that the largest number of midwives agreed that poor network connectivity (72.9%), power failures like loadshedding (82.6%), lack of training among midwives (80.6%), lack of resources (83.2%) and lack of computer and technical skills among midwives (75.5%) will negatively affect the implementation of the digitalized triage system. The midwives further agreed that resistance and resilience to change among midwives (67.1%) and lack of funds (76.8%) will also have a negative effect on the implementation of the digitalized triage system. This highlights that midwives are mindful of the potential challenges in implementing the digitalized triage system.

On the other hand, the majority of the midwives agreed that the implementation of a digitalized triage system will decrease workload and paperwork (76.1%), enhance patient outcomes and care (77.4%), guard against data loss such as loss of files (86.5%) and improve time management and efficiency (85.8%). Furthermore, the midwives agreed that the digitalized triage system will have an influence towards learning new skills (89.7%) and making triaging easier (87.1%). These findings highlight that despite the challenges perceived by the midwives, they also noted a positive impact towards the implementation of a digitalized triage system. Nonetheless, most of the means were 2.51 and above, indicating that the majority of midwives agreed with all the constructs of perceived benefits and effects, and potential implementation challenges. All the factors had a Cronbach’s alpha of greater than 0.6, which suggests that all the factors were considered acceptable and of good reliability to measure the perceptions of midwives of a digitalized triage system [33]. Lastly, the overall Cronbach’s alpha of the 13 items was 0.795, suggesting that the constructs are good and reliable as recommended by Raharjanti et al. [33]. The grouping of the items in the table was achieved by means of factorial analysis, which indicated that only four factors can measure the study objective, supported by the average variance values of the four factors being between 1.084 and 3.850. This suggests that the four factors are appropriate and suitable to measure the perceptions of midwives on the adoption of a digitalized triage system, as they were above 0.5 of the recommended benchmark average variance value as suggested by Shrestha [24]. Prior to the factor analysis, the Kaiser–Meyer Olkin and the Barlett’s test of sphericity were conducted, and were discovered to be 0.767 and <0.001, respectively. This means that the data met eligibility criteria for factor analysis and were suitable and appropriate for factorability.

### 3.4. Mixed-Methods Findings

The primary objective of the study was to investigate the perspectives of midwives on the adoption of a digitalized triage system in South African maternity units. The integration of the data from qualitative and quantitative phases generated a total of four themes, with two expanded and two confirmed findings. The use of the mixed-methods design allowed the authors to expand the study. The authors were able to widen the inquiry into the phenomenon of interest with sufficient depth and breadth of data. The authors first used the qualitative strand to provide depth in the research inquiry to gain rich insights and understanding of the phenomenon of interest. Secondly, they used the quantitative strand to support the qualitative data at a larger population parameter, allowing for a generalization of the findings. This further allowed the authors to bring together a comprehensive understanding of the phenomenon of inquiry. Each method vigorously obtained data and multiple meanings, and the findings were then brought together to provide a comprehensive, complete and context-rich conclusion and understanding of the phenomenon under study [18,26]. The findings are presented in Table 4, below.

## 4. Discussion

The study revealed that midwives have diverse perspectives on the adoption of the digitalized triage system. The midwives’ perceptions aligned with the perceived usefulness and perceived ease of use components of TAM, thus influencing the attitude towards technology adoption. On the other hand, the midwives showed a concern with the existence of external factors that hinder the effective adoption of the digitalized triage system. This is discussed further below.

### 4.1. Availability of Resources and Potential Behavioral Challenges

The integrated findings highlighted anticipated implementation challenges regarding the adoption of a digitalized triage system. This includes challenges such as lack of resources, funds, training and resistance to change. Although these were perceived challenges of a potential digital triage system, this was anticipated acknowledging that SA is middle income country working towards achieving and aligning with the global digital health strategy. However, these study findings were consistent with the studies of Novakowski et al. [34] and Reynolds et al. [35], who reported that digital triage systems have robust implementation challenges like network connectivity, financial support and resources. The midwives in this study further indicated that human resources are also a major requirement for the effective implementation for a digitalized system. This is mainly because a labor ward is busy clinical unit and is unpredictable. Therefore, they need sufficient staff and the staff should be trained to be competent with the digitalized system. This requires an actionable strategy that is aimed at contextualizing digital health interventions by looking at the context in which they will be implemented. The study results suggest that the design and development of digital triage systems should be aligned with the principles of user-centered designs that guide the development and contextualization of digital interventions. When the principles are applied in the digital health sector, they can improve and enhance the usability, acceptability and impact of technology-enabled care [36]. This was supported by the study findings of McCool et al. [37], which indicated that the success and sustainability of a digital health intervention is influenced by the context in which it is to be implemented. Thus, it is essential to establish partnerships with local stakeholders from the initial phase to ensure that the interventions are appropriate and acceptable and that the local funders will continue to support the digital interventions.

Other implementation challenges identified by the study were lack of training and digital illiteracy. Some scholars highlighted that digital illiteracy is associated with a lack of training in digital technologies, resulting in confusion and resistance to its adoption [35]. This aligns with the study findings, as the midwives in this study suggested they should be trained prior to implementation of the digital triage system. The concerns around training certainly necessitate the need for training among the midwives. One of the midwives passionately stated that they are not currently using computers. Therefore, she perceives that to implement a digitalized system, midwives should be trained in computer courses. It seems like digital health interventions like digital triage require strong support through vigorous training to improve the midwives’ knowledge and literacy in digital technology utilization [38]. Novakowski et al. [34] concurs with Zegeye and Parajuli et al. that the lack of training leads to behavioral challenges such as resistance to change, but with time and training a behavioral change regarding the digital interventions is notable [38,39].

On the other hand, just like the study by Zegeye [38], the empirical findings revealed that the midwives are concerned with the sustainability and maintenance of the digitalized triage system. This was also supported by the concerning issue of technical failures and virus attacks. Zegeye states that critical aspects like poor maintenance affect the sustainability and usability of digital health interventions. This includes problems like troubleshooting and other technical errors [38]. One could conclude that such concerning issues require special considerations when developing a digital triage system, noting that midwives are knowledgeable about the biomedical sphere of science and not the technology sphere. This will introduce problems of poor compliance and widespread adoption of the digitalized triage system. Kaboré et al. suggested that the sustainability and maintenance of digital health interventions is complexed and it is influenced by barriers such as limited resources, finances and lack of expertise in human resources [40]. According to the TAM, these findings are external factors that may influence the adoption of the triage system. The TAM highlights that those external factors indirectly affect perceived usefulness and perceived ease of use by midwives [15]. This indicates that to sustain a digital health intervention, there is a dire need to pay attention to such outstanding barriers and establish collaboration with local stakeholders to ensure scalability and sustainability.

### 4.2. Expected Advantages in Usability

Over the past few years, accelerated advancements in digital health technology have prompted changes in human health management. The application of digital health technologies and physical health status and experiences have set the stage for radical progress in individual health and medical management [41]. Similar to existing evidence, the integrated findings highlighted the perceived advantages of usability of a digital triage system. The midwives perceived that the use of a digitalized triage system can prevent loss of information, make triaging easier and improving time management and efficiency. This was also shown by the enthusiasm and relentless excitement and happiness among the midwives. The findings suggest that the midwives have clear visions of what digitalized triage could bring to the triaging of the pregnant women. Positive perceptions of digitalization by midwives indicate positive behavior toward the acceptance of technology use in the provision of care.

According to Abernethy et al., digital health technologies have broadly encompassed the electronic capture of data, along with technical and communication infrastructure [41]. The study by Okolo et al. is consistent with the midwives’ perspectives: the use of digital health technologies ensures proper record keeping, minimizes reliance on paper and reduces errors [42]. The qualitative midwives’ responses highlighted that a digitalized system will enhance easy access to records of care to inform clinical decision making on patient care, reducing delays in interventions to patient and improving patient safety and care. Okolo et al. indicate that digital health technologies with health information technology systems facilitate workflow streamlining and provide healthcare professionals with quick access to patient comprehensive data at point of care [42], thus enabling informed decision making and improved patient outcomes. Anecdotally, one could conclude that a digitalized triage system will assist in the provision of comprehensively available clinical data that can play a crucial role in triage decision making among the midwives. For this effect, it is essential for the South African Ministry of Health to harness the potential of digital health information systems in public healthcare facilities to scale up informed clinical decision making among healthcare professionals. This would therefore reduce maternal and perinatal mortalities associated with preventable factors such as delayed recognition of obstetric emergencies due to lack of comprehensive clinical data to make informed decisions.

### 4.3. Connectivity and Accessibility

The integrated findings indicated that poor network connectivity and power failure are likely to negatively impact the implementation of a digitalized triage system. Similar to the review by Ngwenya et al., these implementation challenges were anticipated mainly because SA is a middle-income country still battling poverty and is also currently battling the issue of loadshedding. It is highlighted by several studies that the issue of power failure also has a direct influence on network connectivity [10]. For instance, a dissertation by Ngwenya indicated that power outages negatively influence the accessibility of digital health technologies due to poor networks. This concern was similar to the qualitative narratives of the midwives in this study; they voiced in depth their concerns around poor network connectivity, particularly during loadshedding [43]. Another thing was that during those power outages, their back-up system often malfunctions. According to Laher et al., power failures and lack of contingency plans contribute to catastrophic events in the healthcare context [44]. Laher et al. further suggested that the issue of power outages has an impact beyond network connectivity; in the clinical environment, it limits patient movements due to malfunctioning elevators and failure to maintain medications [44], thus impacting patient outcomes.

On the other hand, the midwives in this study related the issue of poor network connectivity to the issue of affordability. The midwives’ worry was that once the triage system is digitalized and implemented, how will they afford data for internet connection? This clearly highlights a crucial interoperability challenge around the potential digitalized triage system. In support of the findings, some scholars indicated that poor internet connection, particularly in rural healthcare facilities, is a significant barrier to the use of digital health interventions [45]. The study underscores that the midwives were able to visualize the impact a digitalized triage system might bring into clinical practice. But they could not help but to worry about the impact lack of costs and funds might have on its sustainability and maintenance. The worry about sustainability is valid. However, the crucial learned element is that it can be assumed that the worry about sustainability among the midwives shows willingness towards the long-term use of a digitalized triage format. Thus, the National Digital Health Strategy for South Africa supports the findings, saying that there is a need to establish a robust physical and network infrastructure to address network and internet connectivity constraints that hinder the digital health efforts in South Africa [46].

### 4.4. Potential Impact on Patient Outcomes and Practice

In pursuit of the understanding and perceptions of midwives around the digitalization of a midwife-led triage system, the integrated findings confirmed the potential impact of the digitalized system in patient care and clinical practice. This includes improved patient outcomes, decreased workload and professional development. These findings were consistent with some of the findings of Imison et al. [47], which indicated that digital health technologies like a digitalized triage system improve patient outcomes because they support long-term health management. Thus, midwives will spend more time in their core competencies like treating patients and will have access to all the information they need at their convenience. The midwives expressed that among the improved patient health outcomes, digitalization of the triage system is a wise step towards improving patient safety. The midwives believed that the transition to a digitalized triage system will play a pivotal role in assisting them in the early detection and management of clinical problems affecting pregnant women in a timely manner. They viewed a digitalized triage system as a tool that has the potential to change the way care is provided, as a quality care improvement strategy. This was consistent with the study findings of Perakslis and Ginsburg, which highlighted that digital health technologies are imperative in identifying health risks and in the diagnosis and treatment of pregnant women, consequently, reducing delayed interventions and improving maternal and perinatal outcomes [48]. Digital triage interventions have revolutionized the way patient care is provided; the study by Novakowski et al. further indicated that they have robust potential towards the provision of emergency care and will enrich midwives with full-bodied knowledge and improve their confidence [34]. This was no different to the integrated findings that highlighted that the digitalized tirage system will assist the midwives in learning new skills and will sharpen their knowledge towards patient care. Moreover, from the understanding of the midwives, a digitalized triage system is a big step towards improving the care provided to patients. A study by Kowatsch and Fleisch concurs with this study that digital health interventions are appropriate measures towards improving disease prevention and management [49]. Drawing from the overall integrated findings, one could infer that this calls for a standardized digital triage system. It seems like the implementation of digital triage systems in SA will assist towards attaining the SDG 3 targets, reducing maternal and perinatal mortalities. This aligns well with the WHO framework for quality maternal and child health, which states that digital health interventions like digital triage form part of the actionable information health systems that enables the early use of information to design an appropriate plan of action to improve the care of women and newborns [50]. Drawing from the TAM, it seems like the midwives have a positive attitude towards the proposition of the digitalized triage system and they perceived it as being useful in patient care. For instance, the midwives perceived the triage system as an innovation of change that will enhance patient outcomes and safety [15]. This study’s findings seem to agree with the TAM in that the perceived usefulness of a digitalized triage system has influenced their attitudes towards the adoption of such a proposed digital triage intervention. In addition, the positive attitude towards the proposed intervention is influenced by their current conditions and experience of not having a standardized triage system [15]. This study underscores that researchers and policy makers should draw inferences from findings like this for the development of digital health interventions, putting the midwives at the center, since they are the end-users.

Drawing from the overall findings and existing literature, this allows the conclusion that the use of digital technologies, including digital triage, in the provision of emergency care is beyond the inevitable. The present findings indicate that there is a significant need for policy changes in resources use and allocation. For the successful adoption of digital health interventions like digital triage, this study recommends the employment of a task-shifting approach and decentralization of available resources to facilitate the effective use of digital technology in optimizing care. Policies should take into account existing clinical environments to properly facilitate digital health intervention adoption in maternity units. It is imperative that the stakeholders and government liaise and collaborate with other countries that have successfully implemented a unified digital health strategy to benchmark the optimal adoption and use of digital triage. Moreover, as confirmed by findings, social behavior has an impact on the perception of use. Therefore, an improvement strategy should be considered to empower adopters and users of digital health interventions like digital triage in using such interventions in the provision of quality maternal care.

Lastly, this study was only conducted in district hospitals of Mpumalanga province. Despite similarities being found with other studies, due to contextual limitations, the findings in this study only reflect the perceptions of midwives on the adoption of a digitalized triage systems in the maternity units of the selected hospitals. Thus, the findings can only represent their views on the adoption of a digitalized triage system and not any other digital health technology. Therefore, more research studies should be conducted in different contexts and regions, exploring in depth the perspectives of midwives on the adoption of digital triage systems.

## 5. Conclusions

In conclusion, this study highlighted that midwives in this modern era are accepting of digital health interventions as they perceive them to be useful in improving patient care and their own abilities. However, the midwives also identified potential obstacles to adopting a digitalized triage system. Among the challenges were digital illiteracy, lack of resources and internet connectivity failures. Therefore, the study recommends the development of guidelines and a conceptual framework dedicated to facilitating the widespread implementation of digital triaging in maternity units in South Africa. Stakeholders and policymakers should be consulted and involved in the development of the context-specific guidelines looking at the South African research priorities.

## Figures and Tables

**Table 1:** Themes and subthemes.

Perceived benefits and effects of digitalization of a triage system	1.1. Perceived as easy to use
	1.2. Reduced workload and paperwork
	1.3. Training and professional development opportunities
	1.4. Time efficient and time management
	1.5. Safeguard and prevent information or records loss
	1.6. Improved patient care and outcomes
2. Perceived difficulties with the use of digitalized triage system.	2.1. Concerns about network connectivity and technical failures related to power outages
	2.2. Resilient and resistance to change and digital illiteracy among veteran midwives
	2.3. Concerns about the finances and cost to sustain the digitalized triage system
	2.4. Concerns of training midwives about the reformed digitalized triage
	2.5. Availability of resources

**Table 2:** A joint display delineating qualitative themes and subthemes of the developed questionnaire.

Theme 1: Perceived benefits and effects of digitalization of a triage system (6 items)	❖ Workload and paperwork will decrease with the implementation of the digitalized triage system.
	❖ I will learn new skills with digital triage
	❖ I think patient outcomes and care will both be enhanced by the digitalized triage system.
	❖ The digitalized triage system will guard against data loss (loss of files, for example).
	❖ Improved time management and efficiency will result from the digitalized triage method.
	❖ It will make triaging easier for us
Theme 2: Perceived difficulties with the use of a digitalized triage system (7 items)	❖ Poor network connectivity will affect implementation of digitalized triage system
	❖ Power failure like loadshedding will affect the implementation of digitalized triage system
	❖ Lack of training among midwives will affect the implementation of the digitalized triage system
	❖ Lack of resources will make digitization ineffectual.
	❖ Lack of computer and technical skills among midwives will affect its implementation
	❖ Resistant and resilience to change among midwives will affect its implementation
	❖ Lack of funds and costs will affect its implementation

**Table 3:** Quantitative findings.

Practice	Disagree N (%)	Uncertain N (%)	Agree N (%)	Mean ± SD	Total
**Availability of resources and potential behavioral challenges**					
Lack of training among midwives will affect the implementation of the digitalized triage system	13 (8.4)	17 (11)	125 (80.6)	2.72 ± 0.609	155
Lack of resources will make digitization ineffectual.	9 (5.8)	17 (11)	129 (83.2)	2.77 ± 0.541	155
Lack of computer and technical skills among midwives will affect its implementation	11 (7.1)	27 (17.4)	117 (75.5)	2.68 ± 0.600	155
Resistant and resilience to change among midwives will affect its implementation	25 (16.1)	26 (16.8)	104 (67.1)	2.51 ± 0.759	155
Lack of funds and costs will affect its implementation	18 (11.6)	18 (11.6)	119 (76.8)	2.65 ± 0.680	155
***Cronbach’s alpha***	*0.773*				
**Expected advantages in usability**					
The digitalized triage system will guard against data loss (loss of files, for example).	6 (3.9)	15 (9.7)	134 (86.5)	2.83 ± 0.472	155
Improved time management and efficiency will result from the digitalized triage method.	4 (2.6)	18 (11.6)	133 (85.8)	2.83 ± 0.439	155
It will make triaging easier for us	3 (1.9)	17 (11)	135 (87.1)	2.85 ± 0.408	155
***Cronbach’s alpha***	*0.732*				
**Connectivity and accessibility**					
Poor network connectivity will affect implementation of digitalized triage system	8 (5.2)	34 (21.9)	113 (72.9)	2.68 ± 0.569	155
Power failure like loadshedding will affect the implementation of digitalized triage system	8 (5.2)	19 (12.3)	128 (82.6)	2.77 ± 0.529	155
***Cronbach’s alpha***	*0.676*				
**Potential impact on patient outcomes and practice**					
Workload and paperwork will decrease with the implementation of the digitalized triage system.	14 (9.0)	23 (14.8)	118 (76.1)	2.67 ± 0.636	155
I will learn new skills with digital triage	5 (3.2)	11 (7.1)	139 (89.7)	2.86 ± 0.428	155
I think patient outcomes and care will both be enhanced by the digitalized triage system.	4 (2.6)	31 (20.0)	120 (77.4)	2.75 ± 0.491	155
***Cronbach’s alpha***	*0.604*				
**Overall alpha**	**0.795**				

N = frequency, % = percentage.

**Table 4:** Data integration of the narrative and numeric data.

Principal Themes	Qualitative Results	Quantitative Results
Availability of resources and potential behavioral challenges	The midwives expressed their concern towards implementation challenges such as lack of training, funds and resources. Another midwife expressed their worries towards sustainability and maintenance. Supporting quotes: *“As long as there will be a training on how to use it, everything will be fine.” **P15*** *“…now the patient will be managed on time. So, even if I think it’s expensive. It’s expensive, but it’s good…” **P8***	In total, 67–83% of the midwives agreed that a digitalized triage system is predisposed to potential challenges such as lack of training, lack of resources, resistance to change and lack of funds.
**Mixed-methods meta-inferences**	**Expanded.** The midwives indicated that besides the lack of funds, resources and resistance to change, there are other pressing concerns. The midwives highlighted their concerns of susceptibility of the digitalized triage system to viruses which might cause it to crash and result in a loss of information. Another concern was long-term use and the maintenance and sustainability of the digitalized triage system. Furthermore, their worry with regard to maintenance was that the artifact will require an upgrade to a new version every now and then.	
Expected advantages in usability	The midwives discussed their perceived advantages of implementing a digitalized triage system. Some of the midwives said *“Yes learning, we will get to learn new things.” **P5*** *“I think that it is going to help in reducing the time that we spent on paperwork.” **P15*** *“I think it will prevent loss of records, because sometimes you found out that patient records are lost by that time you already wrote down everything in the records. But at the end of the day, you look for the file and is nowhere to be found. Now there’s a litigation but you can’t find the file anymore” **P2***	Time efficiency and management, prevention of information loss and ease of use were perceived as the expectant advantages of usability of a digitalized triage system, indicated by a total percentage ranging from 85 to 87%. This was also indicated by a mean range of 2.83–2.85, suggesting that most midwives agreed.
**Mixed-Methods meta-inferences**	**Confirmed.** The quantitative results confirmed that time efficiency and management, safeguarding against information loss and ease of use were the most perceived advantages of usability of the digitalized triage system. Some of the midwives indicated that the triage system will assist with the keeping of records for long periods of time and that way, this will facilitate the coordination of care even after the patient comes back after some years.	
Connectivity and accessibility	The midwives revealed that connectivity and accessibility might be an issue in implementing the potential triage system, as this might be hindered by the power failures and poor internet connection. *“…another challenges the digital will bother us when it comes to loadshedding, our electricity it on and off every now and then…Also the backup in our institution is malfunctioning and it delays sometimes, so that it will affect the provision of care particularly when there’s loadshedding and network connectivity issues…” **P9***	In total, 73–83% of the midwives agreed that some of the potential implementation challenges might be poor network connectivity and power failures. This was also indicated by the higher means and standard deviations, ranging between 2.68 and 2.77 and between 0.529 and 0.569.
**Mixed-Methods meta-inferences**	**Confirmed.** The quantitative survey managed to generalize positively that poor network connectivity and power failures and outages will negatively impact the implementation of a digitalized triage system. Power failures are strongly linked to loadshedding and malfunctioning backup generators in clinical institutions.	
Potential impact on patient outcomes and practice	The midwives shared their perceptions on the potential impact of a digitalized triage system in patient care and clinical practice. *It would be okay because it would cut off the paperwork time because we do get tired with handwriting files. So, when we get used to it, I think it’s going to be okay.” **P18*** *“…also, maybe it will also be a way for us to learn, because we won’t be stuck in an old system because we are lazy to learn; so, it is part of learning” **P19***	In total, 76–90% of the midwives responded by agreeing that digitalization of the triage system will reduce workload and paperwork, assist in professional development and enhance patient outcomes.
**Mixed-Method meta-inferences**	**Expanded.** The qualitative responses expanded with the quantitative surveys to state that digitalization of the triage will reduce workload and enhance patient outcomes. One midwife further expressed that it would assist towards reducing errors and improving patients’ safety. The midwives further expressed that the digitalized triage system will reduce maternal and perinatal complications through provision of timely interventions.	

## Data Availability

Data available on reasonable request.

## References

[1] Balfour L. (2020). Implementation and Evaluation of a Clinical Pathway for Non-Invasive Ventilation in Critical Care: A Person-Centred Practice Development Approach.

[2] Cantor A.G., Jungbauer R.M., Totten A.M., Tilden E.L., Holmes R., Ahmed A., Wagner J., Hermesch A.C., McDonagh M.S. (2022). Telehealth strategies for the delivery of maternal health care: A rapid review. Ann. Intern. Med..

[3] Till S., Mkhize M., Farao J., Shandu L.D., Muthelo L., Coleman T.L., Mbombi M., Bopape M., Klingberg S., van Heerden A. (2023). Digital health technologies for maternal and child health in Africa and other Low-and middle-income countries: Cross-disciplinary scoping review with Stakeholder consultation. J. Med. Internet Res..

[4] Makhene A. (2014). Development, Implementation and Evaluation of a Programme to Facilitate Critical Thinking in Nursing Education. Doctoral Dissertation.

[5] Tambo E., Madjou G., Mbous Y., Olalubi O.A., Yah C., Adedeji A.A., Ngogang J.Y. (2016). Digital health implications in health systems in Africa. Eur. J. Pharm. Med. Res..

[6] Van Velthoven M.H., Cordon C. (2019). Sustainable adoption of digital health innovations: Perspectives from a stakeholder workshop. J. Med. Internet Res..

[7] Bhavnani S.P., Narula J., Sengupta P.P. (2016). Mobile technology and the digitization of healthcare. Eur. Heart J..

[8] Van Pelt S., Massar K., Shields-Zeeman L., De Wit J.B., Van der Eem L., Lughata A.S., Ruiter R.A. (2021). The development of an electronic clinical decision and support system to improve the quality of antenatal care in rural Tanzania: Lessons learned using intervention mapping. Front. Public Health.

[9] Wunsch G., Da Costa C.A., Righi R.R. (2017). A semantic-based model for triage patients in emergency departments. J. Med. Syst..

[10] Ngwenya M.W., Muthelo L., Rasweswe M.M., Mothiba T.M. (2025). Leveraging of digital triage to enhance access in obstetric emergencies in the maternity units: A scoping review of digital triage interventions in healthcare. Digit. Health.

[11] von Stillfried D.G. (2021). Toward a digital triage platform for the german healthcare system. Mission–Innov. Telemat. Ehealth High-Defin. Med. Patient-Centered Acute Med..

[12] Engeltjes B., van Herk N., Visser M., Van Wijk A., Cronie D., Rosman A., Scheele F., Wouters E. (2023). Patients’ experiences with an obstetric telephone triage system: A qualitative study. Patient Educ. Couns..

[13] Choosri N., Kungsuwan S. (2023). Feasibility study of using mobile application to support triage and diagnosis clinical decisions for pediatricians: User-centered design approach. Digit. Health.

[14] Marangunić N., Granić A. (2015). Technology acceptance model: A literature review from 1986 to 2013. Univers. Access Inf. Soc..

[15] Davis F.D., Al-Suqri M.N., Al-Aufi A.S. (1989). Technology acceptance model: TAM. Information Seeking Behavior and Technology Adoption.

[16] Taiwo A.A., Downe A.G. (2013). The theory of user acceptance and use of technology (UTAUT): A meta-analytic review of empirical findings. J. Theor. Appl. Inf. Technol..

[17] Lai P.C. (2017). The literature review of technology adoption models and theories for the novelty technology. JISTEM-J. Inf. Syst. Technol. Manag..

[18] Schoonenboom J., Johnson R.B. (2017). How to construct a mixed methods research design. Koln. Z. Fur Soziologie Und Sozialpsychologie.

[19] Polit D.F., Beck C.T. (2018). Essentials of Nursing Research: Appraising Evidence for Nursing Practice.

[20] Rechberg I.D. (2018). Knowledge Management Paradigms, Philosophical Assumptions: An Outlook on Future Research.

[21] Shannon-Baker P. (2016). Making paradigms meaningful in mixed methods research. J. Mix. Methods Res..

[22] Ngwenya M., Muthelo L., Rasweswe M., Mothiba T. (2025). Exploring the Understanding and Triage Practices of Midwives Working in District Hospitals of Mpumalanga Province, South Africa. Open Public Health J..

[23] Naeem M., Ozuem W., Howell K., Ranfagni S. (2023). A step-by-step process of thematic analysis to develop a conceptual model in qualitative research. Int. J. Qual. Methods.

[24] Shrestha N. (2021). Factor analysis as a tool for survey analysis. Am. J. Appl. Math. Stat..

[25] Reeping D., Taylor A.R., Knight D.B., Edwards C. (2019). Mixed methods analysis strategies in program evaluation beyond “a little quant here, a little qual there”. J. Eng. Educ..

[26] Schoonenboom J., Johnson R.B. (2021). The case comparison table: A joint display for constructing and sorting simple tables as mixed analysis. The Routledge Reviewer’s Guide to Mixed Methods Analysis.

[27] Creamer E.G. (2017). An Introduction to Fully Integrated Mixed Methods Research.

[28] Creswell J.W., Clark V.L. (2017). Designing and Conducting Mixed Methods Research.

[29] Younas A., Zeb H., Aziz S.B., Sana S., Albert J.S., Khan I.U., Inayat S., Khan F.H., Rasheed S.P. (2019). Perceived challenges of nurse educators while teaching undergraduate nursing students in Pakistan: An exploratory mixed-methods study. Nurse Educ. Today.

[30] Stahl N.A., King J.R. (2020). Expanding approaches for research: Understanding and using trustworthiness in qualitative research. J. Dev. Educ..

[31] Shi J., Mo X., Sun Z. (2012). Content validity index in scale development. Zhong Nan Da Xue Xue Bao Yi Xue Ban = J. Cent. South Univ. Med. Sci..

[32] Almanasreh E., Moles R., Chen T.F. (2019). Evaluation of methods used for estimating content validity. Res. Soc. Adm. Pharm..

[33] Raharjanti N.W., Wiguna T., Purwadianto A., Soemantri D., Indriatmi W., Poerwandari E.K., Mahajudin M.S., Nugrahadi N.R., Roekman A.E., Saroso O.J. (2022). Translation, validity and reliability of decision style scale in forensic psychiatric setting in Indonesia. Heliyon.

[34] Novakowski S.K., Kabajaasi O., Kinshella M.L., Pillay Y., Johnson T., Dunsmuir D., Pallot K., Rigg J., Kenya-Mugisha N., Opar B.T. (2022). Health worker perspectives of Smart Triage, a digital triaging platform for quality improvement at a referral hospital in Uganda: A qualitative analysis. BMC Pediatr..

[35] Reynolds C.W., Horton M., Lee H., Harmon W.M., Sieka J., Lockhart N., Lori J.R. (2023). Acceptability of a whatsapp triage, referral, and transfer system for obstetric patients in rural Liberia. Ann. Glob. Health.

[36] Ahmer H., Farooqui K., Jivani K., Adamjee R., Hoodbhoy Z. (2024). Applying the principles for digital development to improve maternal and child health in the Peri-urban areas of Karachi, Pakistan. PLoS Digit. Health.

[37] McCool J., Dobson R., Muinga N., Paton C., Pagliari C., Agawal S., Labrique A., Tanielu H., Whittaker R. (2020). Factors influencing the sustainability of digital health interventions in low-resource settings: Lessons from five countries. J. Glob. Health.

[38] Zegeye R.T. (2023). Strategies to Implement Mobile Health Interventions for Diabetes Management in Ethiopia. Doctoral Dissertation.

[39] Parajuli R., Bohara D., Kc M., Shanmuganathan S., Mistry S.K., Yadav U.N. (2022). Challenges and opportunities for implementing digital health interventions in Nepal: A rapid review. Front. Digit. Health.

[40] Kaboré S.S., Ngangue P., Soubeiga D., Barro A., Pilabré A.H., Bationo N., Pafadnam Y., Drabo K.M., Hien H., Savadogo G.B. (2022). Barriers and facilitators for the sustainability of digital health interventions in low and middle-income countries: A systematic review. Front. Digit. Health.

[41] Abernethy A., Adams L., Barrett M., Bechtel C., Brennan P., Butte A., Faulkner J., Fontaine E., Friedhoff S., Halamka J. (2022). The Promise of Digital Health: Then, Now, and the Future. NAM Perspect..

[42] Okolo C.A., Ijeh S., Arowoogun J.O., Adeniyi A.O., Omotayo O. (2024). Reviewing the impact of health information technology on healthcare management efficiency. Int. Med. Sci. Res. J..

[43] Ngwenya M.W. (2023). Strategies to Enhance the Utilization of Digital Health in Early Detection and Treatment of Pre-Eclampsia by Gravid Women in Emalahleni Local Municipality, Mpumalanga Province. Doctoral Dissertation.

[44] Laher A.E., Van Aardt B.J., Craythorne A.D., Van Welie M., Malinga D.M., Madi S. (2019). ‘Getting out of the dark’: Implications of load shedding on healthcare in South Africa and strategies to enhance preparedness. S. Afr. Med. J..

[45] Badr J., Motulsky A., Denis J.L. (2024). Digital health technologies and inequalities: A scoping review of potential impacts and policy recommendations. Health Policy.

[46] Africa NDoHS (2019). National Digital Health Strategy for South Africa 2019–2024.

[47] Imison C., Castle-Clarke S., Watson R., Edwards N. (2016). Delivering the Benefits of Digital Health Care.

[48] Perakslis E., Ginsburg G.S. (2021). Digital health—The need to assess benefits, risks, and value. JAMA.

[49] Kowatsch T., Fleisch E. (2021). Digital health interventions. Connected Business: Create Value in a Networked Economy.

[50] World Health Organization (2016). Standards for Improving Quality of Maternal and Newborn Care in Health Facilities.

